# Application of Intelligent Paradigm through Neural Networks for Numerical Solution of Multiorder Fractional Differential Equations

**DOI:** 10.1155/2022/2710576

**Published:** 2022-01-19

**Authors:** Naveed Ahmad Khan, Osamah Ibrahim Khalaf, Carlos Andrés Tavera Romero, Muhammad Sulaiman, Maharani A. Bakar

**Affiliations:** ^1^Department of Mathematics, Abdul Wali Khan University, Mardan, KP, Pakistan; ^2^Al-Nahrain Nanorenewable Energy Research Center, Al-Nahrain University, Baghdad 10001, Iraq; ^3^COMBA R&D Laboratory, Faculty of Engineering, Universidad Santiago de Cali, 76001 Cali, Colombia; ^4^Special Interest Group Modelling and Data Analytics, Faculty of Ocean Engineering Technology and Informatics, Universiti Malaysia Terengganu, Kuala Nerus, Terengganu 21300, Malaysia

## Abstract

In this study, the intelligent computational strength of neural networks (NNs) based on the backpropagated Levenberg-Marquardt (BLM) algorithm is utilized to investigate the numerical solution of nonlinear multiorder fractional differential equations (FDEs). The reference data set for the design of the BLM-NN algorithm for different examples of FDEs are generated by using the exact solutions. To obtain the numerical solutions, multiple operations based on training, validation, and testing on the reference data set are carried out by the design scheme for various orders of FDEs. The approximate solutions by the BLM-NN algorithm are compared with analytical solutions and performance based on mean square error (MSE), error histogram (EH), regression, and curve fitting. This further validates the accuracy, robustness, and efficiency of the proposed algorithm.

## 1. Introduction

Mathematicians have regarded the theory of fractional calculus as a branch of pure mathematics for nearly three centuries. However, several researchers have recently discovered that noninteger derivatives and integrals are more useful for modelling phenomena with inherited and memory properties than integer orders [1–4]. Fractional differential equations (FDEs) are used to model various problems in science, engineering, economics, biological sciences, and applied mathematics [5–8]. FDEs are more complex than their integer order since the fractional operators are nonlocal and have weakly singular kernels [9–13]. The complications in integer order introduce significant computational difficulties for numerical methods to obtain solutions for such equations.

Fractional differential equations have wide application in the fields of science and engineering. Some recent applications include fractional-order financial systems [14], electrical circuits [15], nuclear magnetic resonance [16], fractional-order Bloch system [17], fractional-order Lorenz system [18], hepatitis B disease in medicine [19], pollution levels in a lake [20], and fractional-order Chua's system [21]. Due to the high usage of FDEs, several numerical and analytical methods have been proposed. Bhrawy [22–24] uses spectral methods based on Jacobi, Chebyshev, and Legendre polynomials over a bounded domain for an approximate solution of FDE's. Atabakzadeh [25] and Tripathi [26] use the operational matrix of Caputo fractional-order derivatives for Chebyshev polynomials and fractional integration of the generalized hat basis functions to solve systems of FDEs. Baleanu [27] in 2013 uses modified generalized Laguerre collocation methods and the Tau method based on semi-infinite interval to calculate the approximate solution for linear and nonlinear FDEs. Ahmadian [28, 29] applied the Jacobi operational matrix to study a class of linear fuzzy FDEs. The spectral approximation method is used by Li [30] to compute the fractional derivative and integral and also presents the pseudo-spectral approximation technique for some classes of FDEs. Esmaeili [31] developed a numerical technique in which the properties of the Caputo derivative were used to reduce the fractional differential equation into a Volterra integral equation.

Recently, the use of spectral methods to solve various types of differential and integral equations has gained interest due to their wide applicability in both finite and infinite domains [32–35]. These methods include Galerkin [33], collocation [36, 37], Tau [38], and Petrov Galerkin [39] classes. Researchers have widely used the Homotopy perturbation method (HPM) [40, 41], Legendre wavelets method (LWM) [42, 43], fractional-order Laguerre and Jacobi Tau methods [44, 45], Chebyshev Tau method (CTM) [45], variational iteration method (VIM) [46], differential transform method (DTM) [40], Bernoulli wavelets method (BWM) [47, 48], and Adomian decomposition method (ADM) [49] for the numerical solution of fractional differential equations.

In recent times, stochastic numerical techniques based on artificial intelligence have been developed to solve stiff nonlinear problems arising in various fields. Such stochastic computing techniques use artificial neural networks to model approximate solutions. These numerical solvers have wide applications in various fields including petroleum engineering [50], heat transfer [51], civil engineering [52], wire coating dynamics [53], and diabetic retinopathy classification [54]. The abovementioned techniques inspire the authors to explore and incorporate soft computing architectures as an alternative, precise, and feasible way for solving nonlinear multiorder fractional differential equations. The main purpose of this article is to obtain approximate solutions for FDEs using artificial neural networks based on the Levenberg–Marquard algorithm. Some highlighted features of the given study are illustrated as follows:

- Novel applications of neuroheuristic techniques based on backpropagated Levenberg–Marquardt neural networks (BLM-NNs) are presented to obtain numerical solutions for different classes of nonlinear multi-order fractional differential equations.
- The processes of training, validation, and testing are carried out by generating a reference solution or data set by using an analytical solution for different cases and examples of FDEs.
- The performance of the proposed scheme is incorporated by fitting the approximate solutions with the reference solution. The absolute error between the targeted data and approximate solutions illustrates the worth and accuracy of the BLM-NN algorithm.
- Convergence analysis based on mean square errors of the objective function, regression analysis, and histogram plots are employed to study the complexity, robustness, and correctness of the design scheme.
- The advantage of the proposed design is that it does not require any initial parameter settings. It has simple and smooth implementation with exhaustive applicability and stability.

## 2. Solution Methodology

**Figure 1:**
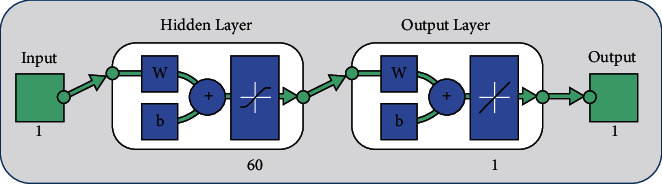
Structure of a supervised neural network.

**Figure 2:**
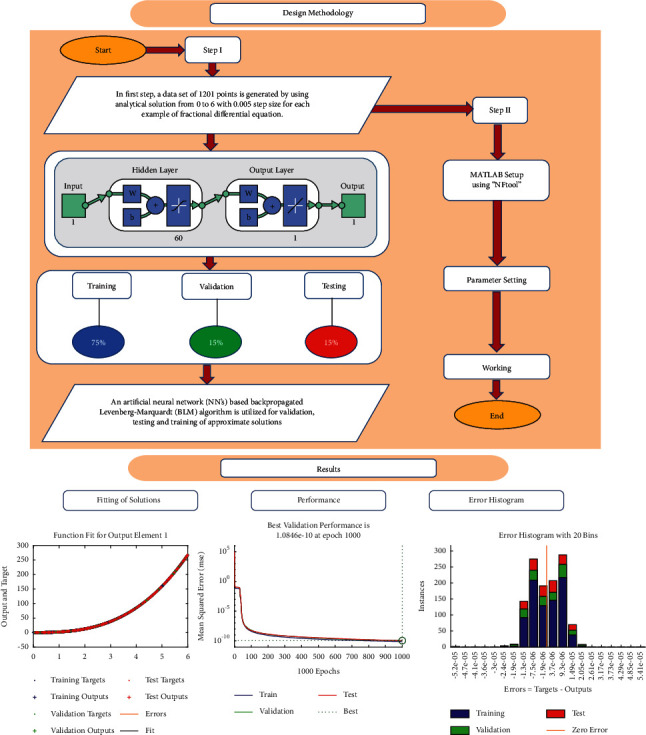
A complete overview of the working procedure of the BLM-NN algorithm.

In the field of artificial intelligence (AI), supervised machine learning refers to a collection of algorithms that describe a predictive model based on data set with known outcomes. The method is learned through the uses of an efficient teaching algorithm, such as artificial neural networks, which use optimization procedures to minimise the error function. The infrastructure of the proposed BLM-NN algorithm is based on two fundamental steps. In the first step, a data set of 1201 points is generated by using an analytical solution from 0 to 6 with a 0.005 step size. In the next step, the Levenberg–Marquardt framework of fitting tool “nftool” from the neural network toolbox of MATLAB R2018a is used to approximate the solutions with 75% training, 15% validation, and 15% testing. The suggested structure of the BLM-NN algorithm with 60 neurons is shown in Figure 1. A summary of the working procedure of the design scheme is presented through the flow chart in Figure 2.

The performance of a design scheme is measured through the performance indicators in terms of mean square error (MSE) of fitness function, regression *R*^2^, error histograms, and absolute errors (AE). The mathematical formulation of the MSE, *R*^2^, and AE is given as follows:

(1) $$\begin{array}{c} \text{MSE}=\frac{1}{k}\sum_{j=1}^{k}{\left(u_{j}\left(t\right)-{\hat{u}}_{j}\left(t\right)\right)}^{2},\\ R^{2}=1-\frac{\sum_{j=1}^{k}{\left({\hat{u}}_{j}\left(t\right)-{\bar{u}}_{j}\left(t\right)\right)}^{2}}{\sum_{j=1}^{k}{\left(u_{j}\left(t\right)-{\bar{u}}_{j}\left(t\right)\right)}^{2}}, \end{array}$$

and

(2) $$\begin{array}{c} \text{AE}=\left|u_{j}\left(t\right)-{\hat{u}}_{j}\left(t\right)\right|,\;j=1,2,\ldots ,k. \end{array}$$

Here, *u*_*j*_, ${\bar{u}}_{j}$, and ${\hat{u}}_{j}$ denote the reference, approximate, and mean of the solution at *j*th input and *k* is the number of mesh points. The desired value of MSE and AE for perfect fitting is equal to zero, while the value of *R*^2^ is one.

## 3. Numerical Experimentation

In order to illustrate the performance of the BLM-NN algorithm, we have considered various examples of nonlinear multiorder fractional differential equations. All calculations and evaluations for this research are performed on HP laptop EliteBook 840 G2 with an intel(R) Core (TM) i5-5300 CPU @ 2.30 GHz, 8.00 GB RAM, 64 bit operating in Microsoft Windows 10 Education edition, running the R2018a version of MATLAB.

**Example 1 .**

Consider the following nonlinear fractional differential equation [55]:

(3) $$\begin{array}{c} D^{\upsilon }u\left(x\right)+u^{2}\left(x\right)=\Gamma \left(\upsilon +2\right)x+\frac{6x^{3-v}}{\Gamma \left(4-\upsilon \right)}+{\left(x^{\upsilon +1}+x^{3}\right)}^{2},\;0<\upsilon \leq 2, \end{array}$$

with the following equation:

(4) $$\begin{array}{c} u\left(0\right)=u^{\prime }\left(0\right)=0. \end{array}$$

The exact solution of (3) is *u*(*x*)=*x*^*υ*+1^+*x*^3^. Four fractional orders are considered i.e., Case I *υ*=1.2, Case II *υ*=1.4, Case III *υ*=1.6, and Case IV *υ*=1.8.

**Figure 3:**
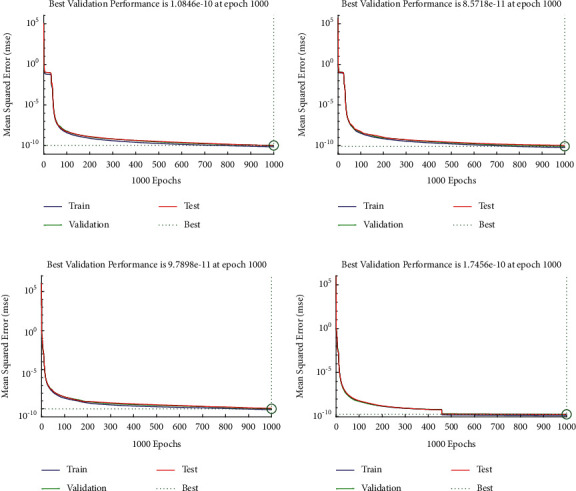
Performance analysis of design schemes based on MSE for different cases of Example 1. (a) Case I, (b) case II, (c) case III, and (d) case IV.

**Figure 4:**
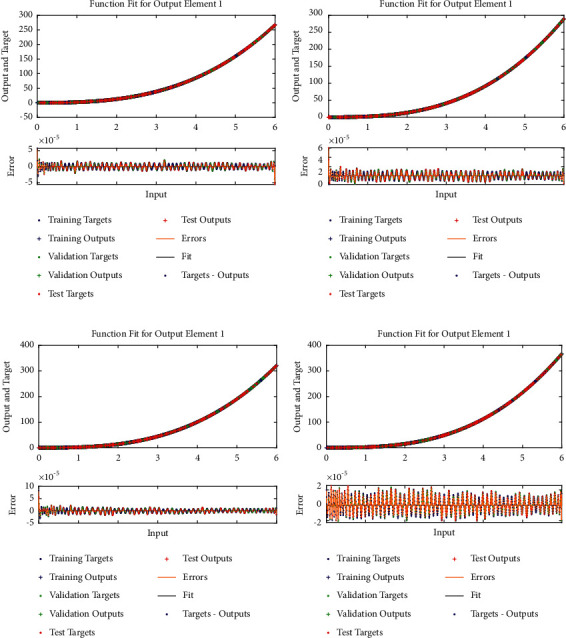
Comparison of the analytical solution with the approximate solution obtained by the BLM-NN algorithm for different orders of FDE as illustrated in Example 1. (a) Case I, (b) case II, (c) case III, and (d) case IV.

**Figure 5:**
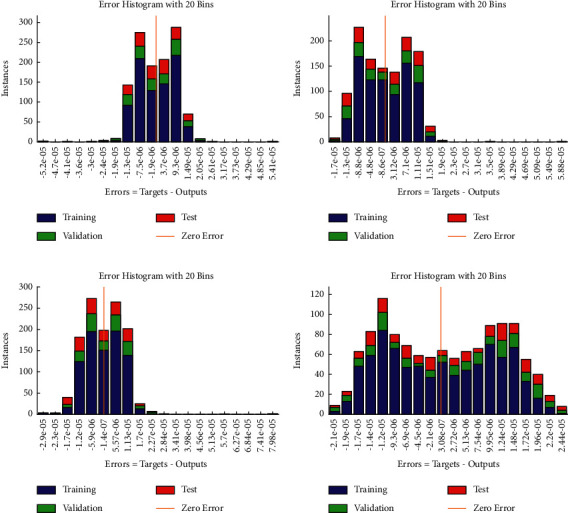
Error histogram between target values and approximated values for multiple orders of equation (4). (a) Case I, (b) case II, (c) case III, and (d) case IV.

**Figure 6:**
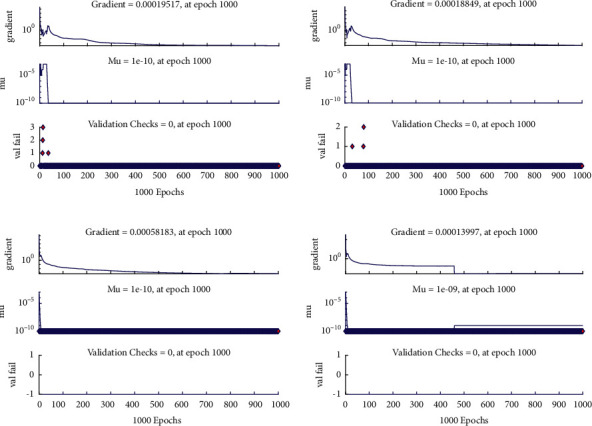
Training state of design scheme for all cases of Example 1. (a) Case I, (b) case II, (c) case III, and (d) case IV.

**Figure 7:**
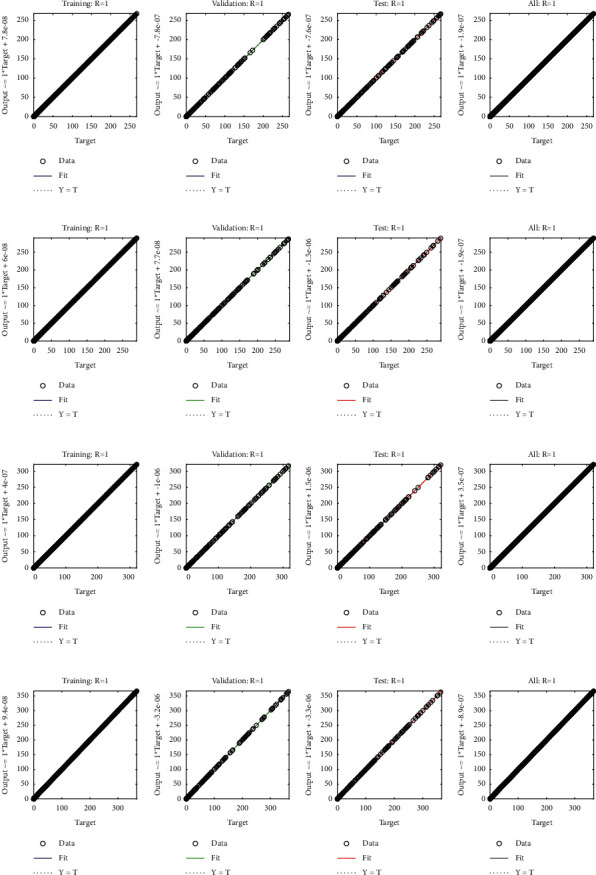
Regression analysis of cases I, II, III, and IV of Example 1.

In order to find approximate solutions for various orders of (3), the BLM-NN algorithm is executed using “nftool” in the MATLAB package. The performance and convergence of the mean square error (MSE) of the objective function are shown in Figure 3. It can be seen that the best validated performance for *υ*=1.2, 1.4, 1.6, and 1.8 are 1.0846*e* − 10, 8.5718*e* − 11, 9.7898*e* − 11, and 1.7456*e* − 10 which are attained at 1000 epoch.  Table 1 demonstrates the approximate solution for each case of Example 1. Further, the fitting of approximate solutions with analytical solutions is plotted in Figure 4. The absolute errors between targeted data and obtained solutions for multiple orders of (3) are illustrated in Figures 4 and 5, respectively. The values of AE for each case lie around 10^−5^ to 10^−6^, 10^−5^ to 10^−7^, 10^−5^ to 10^−7^, and 10^−5^ to 10^−8^, respectively.  Table 2 represents the measure of convergence for each testing, validation, training, gradient, mu, and complexity analysis in terms of time taken by the system to achieve the desired results. It can be seen that the values for the gradient for each case lie around 10^−4^ to 10^−7^, while the maximum time taken by the system is 5 seconds. The training state of operators during the process of optimization for Example 1 is shown in Figure 6. The accuracy and efficiency of the proposed algorithm is shown by the results of regression as dictated in Figure 7.

**Example 2 .**

**Figure 8:**
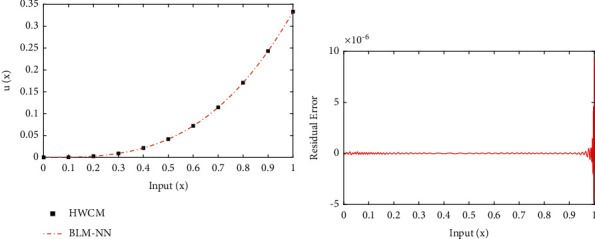
(a) Approximate solutions and (b) absolute errors in our solutions for Example 2.

Consider the following nonlinear multiterm nonhomogenous fractional differential equation as [56]

(5) $$\begin{array}{c} D^{2.2}u\left(x\right)+D^{\alpha_{2}}u\left(x\right)+D^{\alpha_{1}}u\left(x\right)+u^{3}\left(x\right)=\frac{2}{\Gamma \left(1.8\right)}x^{0.8}+\frac{2}{\Gamma \left(4-\alpha_{2}\right)}x^{3-\alpha_{2}}+\frac{2}{\Gamma \left(4-\alpha_{1}\right)}x^{3-\alpha_{1}}+{\left(\frac{x^{3}}{3}\right)}^{3}, \end{array}$$

subjected to the following equation:

(6) $$\begin{array}{c} y\left(0\right)=y^{\prime }\left(0\right)=y^{\prime \prime }\left(0\right)=0, \end{array}$$

where *α*_1_ and *α*_2_ are 0.75 and 1.25, respectively. The exact solution of (5) is *u*(*x*)=*x*^3^/3. The approximate solution obtained by the proposed algorithm for (5) are shown in Figure 8(a). In addition, Figure 8(b) shows the accuracy of the solutions in terms of residual errors. It shows the accuracy of the solutions as the errors are approaching zero. Further, to validate the efficiency, absolute errors in solutions of BLM-NN are dictated through Table 3. It can be observed that the results of the design scheme overlap the exact solutions with minimum absolute errors as compared to the Haar wavelet collocation method [56] and the Bernoulli wavelet operational matrix method [57].

**Example 3 .**

**Figure 9:**
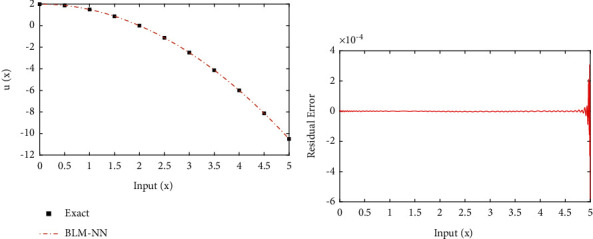
(a) Approximate solutions and (b) absolute errors in our solutions for Example 3.

Consider the following nonlinear multiterm fractional differential equation as follows:

(7) $$\begin{array}{c} aD^{2}u\left(x\right)+b\left(x\right)D^{\alpha_{2}}u\left(x\right)+c\left(x\right)Du\left(x\right)+e\left(x\right)D^{\alpha_{1}}u\left(x\right)+k\left(x\right)u\left(x\right)=f\left(x\right),\;x\in \left[0,T\right], \end{array}$$

where 0 < *α*_1_ ≤ 1,1 < *α*_2_ ≤ 2 and *f*(*x*) are defined as follows:

(8) $$\begin{array}{c} f\left(x\right)=a-\frac{b\left(x\right)}{\Gamma \left(3-\alpha_{2}\right)}x^{2-\alpha_{2}}-c\left(x\right)x-\frac{e\left(x\right)}{\Gamma \left(3-\alpha_{1}\right)}x^{2-\alpha_{1}}+k\left(x\right)\left(2-\frac{1}{2}x^{2}\right), \end{array}$$

with initial conditions as follows:

(9) $$\begin{array}{c} y\left(0\right)=2,\\ y^{\prime }\left(0\right)=0. \end{array}$$

for *a*=1, *b*(*x*)=*x*^1/2^, *c*(*x*)=*x*^1/3^, *e*(*x*)=*x*^1/4^, *k*(*x*)=*x*^1/5^, *α*_1_=0.5,  and *α*_2_=1.5; the exact solution of (7) is *y*(*t*)=2 − 1/2*x*^2^.  Figure 9 depicts the comparison of exact and numerical solutions obtained by the design algorithm for (7). The results calculated by the BLM-NN algorithm are compared with those obtained by the generalized block pulse operational matrix method [58] as shown in Table 4. The absolute errors lie around 10^−7^ to 10^−8^. The values of the performance function in terms of mean square error are shown in Table 2. The results in terms of computational complexity and absolute errors show the accuracy of the proposed algorithm in calculating solutions to fractional differential equations.

**Example 4 .**

Consider the following system of fractional differential equation:

(10) $$\begin{array}{c} D_{C}^{\upsilon }u_{1}\left(x\right)=u_{2}\left(x\right), \end{array}$$

(11) $$\begin{array}{c} D_{C}^{\upsilon }u_{2}\left(x\right)=-u_{1}\left(x\right)-u_{2}\left(x\right)+x^{\upsilon +1}+\frac{\pi \;\csc\left(\pi \upsilon \right)x^{1-\upsilon }}{\Gamma \left(-\upsilon -1\right)\Gamma \left(2-\upsilon \right)}+\frac{\pi x\;\csc\left(\pi \upsilon \right)}{\Gamma \left(-\upsilon -1\right)}, \end{array}$$

with the following equation:

(12) $$\begin{array}{c} u_{1}\left(0\right)=0,\\ u_{2}\left(0\right)=0. \end{array}$$

The exact solutions of (10) and (11) are given as follows:

(13) $$\begin{array}{c} u_{1}\left(x\right)=x^{1+\upsilon },\\ u_{2}\left(x\right)=\frac{\pi \upsilon \left(\upsilon +1\right)\csc\left(\pi \upsilon \right)}{\Gamma \left(1-\upsilon \right)}x. \end{array}$$

We have solved this problem by considering different cases based on orders of derivative, i.e., Case I *υ*=1/4, Case II *υ*=1/2, Case III *υ*=2/3, and Case IV *υ*=9/10.

**Figure 10:**
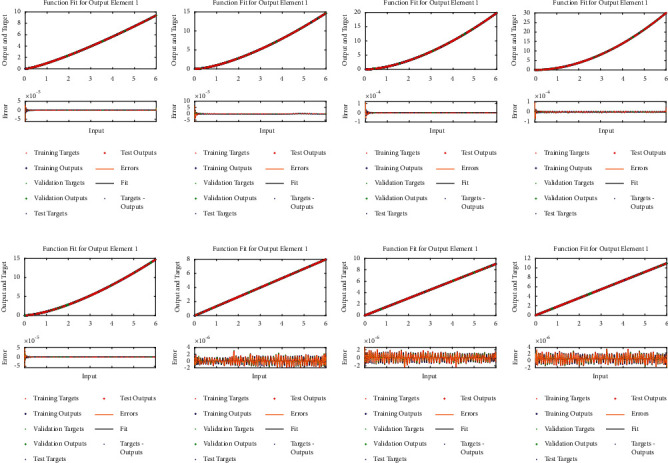
Comparison of analytical solution with approximate solution for *u*_1_(*a*) − (*d*) and *u*_2_(*e*) − (*h*) for multiple orders of Example 4. (a) Case I, (b) case II, (c) case III, (d) case IV, (e) case I, (f) case II, (g) case III, and (h) case IV.

**Figure 11:**
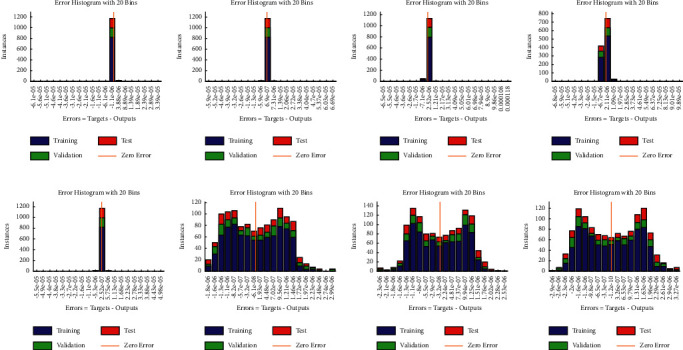
Error histogram between target values and approximated values for multiple orders of equations (10) and (11). (a) Case I, (b) case II, (c) case III, (d) case IV, (e) case I, (f) case II, (g) case III, and (h) case IV.

**Figure 12:**
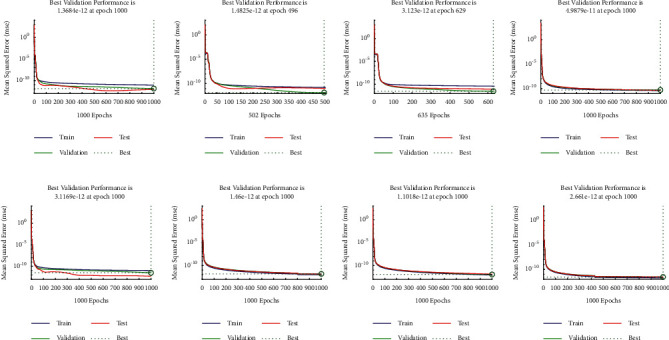
Convergence of performance in terms of mean square error for *u*_1_(*x*) (a–d) and *u*_2_(*x*) (e–h) for multiple orders of Example 4. (a) Case I, (b) case II, (c) case III, (d) case IV, (e) case I, (f) case II, (g) case III, and (h) case IV.

**Figure 13:**
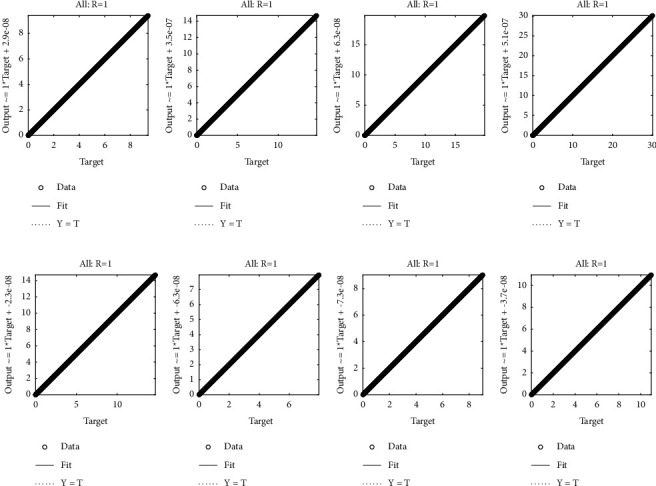
Regression analysis of cases I, II, III, and IV of Example 4. (a) Case I, (b) case II, (c) case III, (d) case IV, (e) case I, (f) case II, (g) case III, and (h) case IV.

Approximate solutions obtained by the BLM-NN algorithm for *u*_1_(*x*) and *u*_2_(*x*) are dictated in Table 5. The comparison or fitting of analytical solutions with approximate solutions is plotted in Figure 10. It can be seen that high-overlapping solutions with a minimum absolute error are obtained.  Figure 11 represents the error histograms for different cases. The values of absolute errors for each case of (10) and (11) lie around 10^−5^ to 10^−6^, 10^−5^ to 10^−7^, 10^−4^ to 10^−6^, 10^−5^ to 10^−6^, 10^−5^ to 10^−7^, 10^−6^ to 10^−8^, 10^−6^ to 10^−8^, and 10^−6^ to 10^−10^, respectively. The smoothness of the algorithm has been detected from the convergence of the mean square error of the objective function.  Figure 12 dictates that validated performance for each case of *u*_1_(*x*) and *u*_2_(*x*) are 1.3684*e* − 12, 1.4825*e* − 12, 3.123*e* − 12, 4.9879*e* − 11, 3.1169*e* − 12, 1.46*e* − 12, 1.1018*e* − 12, and 2.661*e* − 12, respectively. Further, details of performance indices are provided in Table 5. The values of the gradient for each case are 1.84*e* − 07, 3.49*e* − 06, 1.28*e* − 05, 1.43*e* − 07, 2.02*e* − 06, 4.23*e* − 07, 8.03*e* − 07, and 3.33*e* − 07. From Table 6, it can be seen that the values of mu for each case at 1000 epochs lie around 10^−11^ to 10^−13^. Regression analysis shown in Figure 13 further validates the efficiency and correctness of the technique.

## 4. Conclusion

In this paper, we have designed an integrated soft computing technique based on supervised learning. The computational strength of neural networks is utilized by the backpropagated Levenberg–Marquardt (BLM) algorithm to find approximate solutions for nonlinear multi-order fractional differential equations. The working procedure of BLM-NN algorithms is categorized into two steps in which the reference solution is generated by using analytical solutions. Furthermore, the data set is used by the BLM algorithm for validation, testing, and training of approximate solutions. Multiple figures, in terms of approximate solutions, curve fitting of analytical solutions and output data, error histograms, and regression and convergence of performance, are plotted to validate the efficiency of the design scheme. The tabulated data and figures dictate the accuracy, efficiency, and robustness of the design paradigm.

In the future, the authors would like to extend the concept of soft computing based on neural networks to solve the mathematical models represented by partial differential equations and partial fractional differential equations.

## Figures and Tables

**Table 1:** Approximate solutions obtained by the proposed algorithm for different cases of multiorder fractional differential equations.

*x*	Example 1				Example 2	Example 3
	Case I	Case II	Case III	Case IV		
0	0	0	0	0	0	2
0.5	0.342 638	0.314 465	0.289 938	0.268 587	0.041 666 67	1.875
1	2	2	2	2	0.333 333 33	1.5
1.5	5.815 061	6.021 178	6.244 705	6.487 114	1.125	0.875
2	12.594 79	13.278 03	14.062 87	14.964 4	2.666 666 67	0
2.5	23.132 03	24.641 87	26.455 39	28.633 64	5.208 333 33	−1.125
3	38.211 58	40.966 61	44.398 64	48.674 02	9	−2.5
3.5	58.613 01	63.094 17	68.851 28	76.247 64	14.291 666 7	−4.125
4	85.112 13	91.857 62	100.758 3	112.502 9	21.333 333 3	−6
4.5	118.481 9	128.083 1	141.054	158.577	30.375	−8.125
5	159.493 2	172.591 3	190.6632	215.5975	41.666 666 7	−10.5
5.5	208.915	226.198 3	250.5035	284.6834	55.458 333 3	−13.125
6	267.514 9	289.716 2	321.4856	366.9467	72	−16

**Table 2:** Statistical analysis of performance measures including MSE, gradient, mu, number of iterations, and time taken by the system for calculating the results of Examples 1, 2, and 3.

Example	Case	Hidden neurons	Mean square error			Gradient	Mu	Epochs	Regression	Time (s)
			Training	Validation	Testing					
1	I	60	7.50E-11	1.08E-10	1.06E-10	1.00E-07	1.00E-10	1000	1	5
	II	60	5.84E-11	8.57E-11	1.13E-10	1.00E-07	1.00E-10	1000	1	5
	III	60	7.64E-11	9.79E-11	1.15E-10	5.82E-04	1.00E-10	1000	1	5
	IV	60	1.26E-10	1.75E-10	1.66E-10	1.40E-04	1.00E-09	1000	1	5
2		60	5.18E-14	1.12E-13	1.61E-13	1.24E-08	1.00E-13	890	1	2
3		60	5.52E-12	1.58E-13	2.53E-12	1.51E-06	1.00E-10	1000	1	5

**Table 3:** Comparison of absolute errors in solutions obtained by the BLM-NN algorithm with the Bernoulli wavelet operational matrix method and the Haar wavelet collocation method.

*t*	*N* = 08		*N* = 16		*N* = 32		*N* = 64		BLM-NN
	HWCM	BWOM	HWCM	BWOM	HWCM	BWOM	HWCM	BWOM	
1.00E-01	2.27E-04	1.10E-03	6.55E-05	2.00E-04	1.83E-05	6.96E-05	5.05E-06	1.53E-05	6.40E-08
2.00E-01	4.76E-04	1.70E-03	1.33E-04	5.00E-04	3.69E-05	1.17E-04	1.02E-05	3.69E-05	4.82E-08
3.00E-01	6.92E-04	2.50E-03	1.93E-04	8.00E-04	5.34E-05	1.75E-04	1.48E-05	5.42E-05	2.64E-08
4.00E-01	8.72E-04	4.00E-03	2.43E-04	9.00E-04	6.73E-05	2.74E-04	1.87E-05	5.91E-05	3.02E-10
5.00E-01	1.02E-03	5.30E-03	2.83E-04	1.40E-03	7.88E-05	3.52E-04	2.20E-05	9.10E-05	2.98E-08
6.00E-01	1.13E-03	5.90E-03	3.15E-04	1.20E-03	8.79E-05	3.87E-04	2.46E-05	8.28E-05	5.82E-08
7.00E-01	1.21E-03	5.30E-03	3.38E-04	1.70E-03	9.48E-05	3.58E-04	2.66E-05	1.14E-04	2.01E-08
8.00E-01	1.26E-03	5.80E-03	3.54E-04	1.90E-03	9.96E-05	3.96E-04	2.81E-05	1.26E-04	5.93E-08
9.00E-01	1.28E-03	8.00E-03	3.63E-04	1.60E-03	1.03E-04	5.36E-04	2.91E-05	1.12E-04	2.10E-06

**Table 4:** Comparison of absolute errors in the solutions of the BLM-NN algorithm with the generalized block pulse operational matrix method for different step sizes.

*x*	*h* = 0.1		*h* = 0.05	*h* = 0.025	*h* = 0.0125	*h* = 0.00625
	BLM-NN		GBPOM			
0.5	9.81E-08	2.40E-03	6.08E-04	1.52E-04	3.82E-05	9.55E-06
1.5	6.02E-07	2.00E-03	5.06E-04	1.27E-04	3.17E-05	7.95E-06
2.5	4.38E-07	1.60E-03	4.41E-04	1.10E-04	2.76E-05	6.90E-06
3.5	2.69E-07	1.60E-03	4.00E-04	1.03E-05	2.58E-05	6.46E-06
4.5	9.03E-07	1.60E-03	4.00E-05	9.99E-04	2.50E-05	6.25E-06

**Table 5:** Approximate solutions obtained by the proposed algorithm for different cases of the system of FDEs given in Example 4.

*x*	Case I		Case II		Case III		Case IV	
	*u*_1_(*x*)	*u*_2_(*x*)	*u*_1_(*x*)	*u*_2_(*x*)	*u*_1_(*x*)	*u*_2_(*x*)	*u*_1_(*x*)	*u*_2_(*x*)
0.0	0	0	0	0	0	0	0	0
0.5	0.420 448	0.566 502	0.353 553	0.664 67	0.314 98	0.752 288	0.267 943	0.913 678
1.0	1	1.133 003	1	1.329 34	1	1.504 575	1	1.827 355
1.5	1.660 023	1.699 505	1.837 117	1.994 011	1.965 556	2.256 863	2.160 595	2.741 033
2.0	2.378 414	2.266 006	2.828 427	2.658 681	3.174 802	3.009 151	3.732 132	3.654 71
2.5	3.143 584	2.832 508	3.952 847	3.323 351	4.605 039	3.761 439	5.702 772	4.568 388
3.0	3.948 222	3.399 009	5.196 152	3.988 021	6.240 251	4.513 726	8.063 626	5.482 065
3.5	4.787 238	3.965 511	6.547 9	4.652 691	8.068 264	5.266 014	10.807 6	6.395 743
4.0	5.656 854	4.532 012	8	5.317 362	10.079 37	6.018 302	13.928 81	7.309 42
4.5	6.554 139	5.098 514	9.545 942	5.982 032	12.265 56	6.770 59	17.422 23	8.223 098
5.0	7.476 744	5.665 015	11.180 34	6.646 702	14.620 09	7.522 877	21.283 5	9.136 775
5.5	8.422 739	6.231 517	12.898 64	7.311 372	17.137 12	8.275 165	25.508 75	10.050 45
6.0	9.390 507	6.798 019	14.696 94	7.976 042	19.811 56	9.027 453	30.094 52	10.964 13

**Table 6:** Statistical analysis of performance measures including MSE, gradient, mu, number of iterations, and time taken by the system for obtaining the results of Example 4.

Performance measures	Case I		Case II		Case III		Case IV	
	*u*_1_(*x*)	*u*_2_(*x*)	*u*_1_(*x*)	*u*_2_(*x*)	*u*_1_(*x*)	*u*_2_(*x*)	*u*_1_(*x*)	*u*_2_(*x*)
**Hidden neurons**	60	60	60	60	60	60	60	60
**Training**	8.67E-12	1.04E-11	1.64E-11	9.39E-13	3.88E-11	8.32E-13	3.83E-11	1.74E-12
**Validation**	1.37E-12	3.12E-12	1.48E-12	1.46E-12	3.12E-12	1.10E-12	4.99E-11	2.66E-12
**Testing**	8.52E-13	4.94E-13	1.06E-11	1.37E-12	8.76E-12	1.32E-12	4.29E-11	2.66E-12
**Gradient**	1.84E-07	3.49E-06	1.28E-05	1.43E-07	2.02E-06	4.23E-07	8.03E-07	3.33E-07
**Mu**	1.00E-13	1.00E-13	1.00E-13	1.00E-11	1.00E-12	1.00E-12	1.00E-11	1.00E-11
**Epochs**	1000	1000	502	1000	635	1000	1000	1000
**Regression**	1	1	1	1	1	1	1	1
**Time (s)**	6 s	5 s	2 s	5 s	3 s	5 s	5 s	5 s

## Data Availability

The data used to support the findings of this study are available from the corresponding author upon reasonable request.
